# A Fast and Easy Probe Based on CMC/Eu (Ⅲ) Nanocomposites to Detect Acrylamide in Different Food Simulants Migrating from Food-Contacting Paper Materials

**DOI:** 10.3390/polym14173578

**Published:** 2022-08-30

**Authors:** Jiawen Chen, Jun Ye, Mingming Zhang, Jian Xiong

**Affiliations:** 1 State Key Laboratory of Pulp and Paper Engineering, South China University of Technology, Guangzhou 510640, China; 2 School of Food Science and Engineering, South China University of Technology, Guangzhou 510640, China

**Keywords:** CMC/Eu (Ⅲ), fluorescence probe, acrylamide, food contacting material

## Abstract

The residual acrylamide in food paper packaging can be transferred into water and food, which will cause harmful effects on human beings. In this paper, a rapid and easily available fluorescent probe based on carboxymethyl cellulose (CMC)/Eu (Ⅲ) nanocomposites was designed to detect the residue acrylamide with high sensibility. The probe could respond in 1 min. The concentration of acrylamide was linearly correlated to the fluorescence intensity of the probe at the emission wavelength of 615 nm in the concentration range of 0.1–100 μmol/L. The limit of detection (LOD) of the probe was 0.085 μg/L, which is lower than the guideline value of the European Union, the U.S. EPA, and the WHO. An experiment was performed to simulate the acrylamide migrating from food-contacting paper materials to different foods, including waterborne food, alcohol beverage, acidic food, and greasy food. The recoveries and RSDs of acrylamide in all samples indicated that the CMC/Eu (Ⅲ) fluorescent probe was efficient for acrylamide detection. The possible mechanism of the probe for acrylamide detection involved both dynamically quenching and static quenching by forming of non-fluorescent substances.

## 1. Introduction

Polyacrylamide, a linear polymer formed by the radical polymerization of acrylamide monomer, was widely used as a retention aid [1], filter aid [2], and flocculating agent [2,3] in many industries, such as water treatment and papermaking. The application of polyacrylamide in papermaking can improve the quality of paper and reduce the consumption of raw material [1] by increasing the retention rate of fine fibers and fillers [4]. When the paper containing polyacrylamide is used as a food-contact material (FCM), such as in paper cups and paper containers, the migration of the acrylamide monomers to the food is high-risk. Therefore, the residual acrylamide on food-contact materials was strictly limited by the Chinese government.

Acrylamide is genotoxic, neurotoxic, and carcinogenic, and has been proven to cause myasthenia and sensory loss after entering the human body mainly through the digestive system, respiratory system, and skin [5]. Acrylamide has been found to cause tumors [6], neurotoxicity [7], and genotoxicity [8] either in experimental animals, human body, or in vivo and in vitro toxicity tests. In 1994, the International Agency for Cancer Research (IACR) listed acrylamide as a possible human carcinogen [9]. The European Union has established a guideline value of 0.0001 mg/L (0.1 µg/L) for acrylamide in drinking water, and the United States Environmental Protection Agency (US EPA) and the World Health Organization (WHO) set this guideline value to 0.5 µg/L [10,11].

Quantitation of acrylamide is traditionally performed using liquid chromatography/tandem mass spectrometry (LC-MS/MS) [12,13], high-performance liquid chromatography (HPLC) [14,15], gas chromatography (GC) [16], and gas chromatography–mass spectrometry (GC-MS) [17,18]. However, the LOD of these methods is not low enough to meet the requirement of the abovementioned guideline [19]. The Chinese government released a standard method [20] to measure the acrylamide migrating from paper cups, paper food bags, paper wrap, and paper containers with the LOD of 0.01 mg/L.

In the recent years, some new methods with lower LOD have been developed, such as photoelectrochemical (PEC) methods [21], fluorescence sensing detection methods [19], and electrochemical biosensor detection methods [22]. Fluorescence sensing is the most powerful signal transduction mechanism, which has been widely applied because of its simple operation, high selectivity, and sensitivity. For example, A rapid fluorescence method based on gold nanoparticles and carboxyfluorescein (FAM)-labeled double-stranded DNA was proposed, but the responding time was still not short enough [19]. There is also a fluorescence detection measure using Hofmann reaction [23]. However, the synthesis of these probes is complex and not environmentally friendly.

In this study, we used a simple method performed under mild conditions, without using any organic solvent, to synthesize a fluorescent probe derived from CMC/Eu (Ⅲ) nanocomposites for the acrylamide detection. We determined the stability, selectivity, and sensitivity of the fluorescent probe. At the same time, the limit of detection (LOD) of acrylamide in different medium was measured. According to the GB 31604.1-2015, four kinds of food simulants, including waterborne food, alcohol beverage, acidic food, and greasy food, were applied to evaluate the capability of the as-prepared CMC/Eu (Ⅲ) fluorescent probe on the acrylamide detection. The acrylamide migrating from food-contacting paper materials was detected CMC/Eu (Ⅲ) nanocomposites as well. The quenching mechanism of the fluorescent probe by acrylamide was also discussed.

## 2. Materials and Methods

### 2.1. Materials

Acrylamide was purchased from Yousuo Chemical (Heze, Shandong, China). Eu_2_O_3_ was obtained from Yuelong Chemical Industry (Shanghai, China). Maize oil was supplied by Xi Wang Food (Shandong, China). Acetic acid was purchased from Haohua Chemical (Shanghai, China). Citric acid was obtained from Haiping Chemical (Jinan, China). Asparaginate was supplied by Hairui Chemical (Hangzhou, China). Glycine was purchased from Miriel Chemical Technology (Shanghai, China). Paper food bags, paper wraps, paper containers, and paper cups were purchased from a supermarket. All paper products were made by white paper, and there were no extra processing steps for these materials. CMC was from Yingte Chemical Industry (Shijiazhuang, China). Glycine was from Aladdin Biochemical Technology Industry (Shanghai, China). Starch was from Suzhou Deba Chemical Industry (Suzhou, China). Glucose was from Miriel Chemical Technology Industry (Shanghai, China). HCl, TbCl_3_·H_2_O, NaCO_3_, FeCl_3_, BaCl_2_, ZnSO_4_, NaOH, and KCl were from Chemical Reagent Industry (Guangzhou, China). NaHCO_3_ and CaCl_2_ were from Chemical Reagent Industry (Tianjin, China). KBr was from Zhiyuan Chemical Reagent Factory (Tianjin, China).

### 2.2. Methods

#### 2.2.1. Characterization

The morphology of CMC/Eu (Ⅲ) nanocomposites was characterized by EVO18 scanning electron microscope (SEM, Carl Zeiss, Jena, Germany) and a FEI Tecnai G2 Spirit transmission electron microscopy (TEM, FEI Company, Hillsboro, OR, USA). Nano Measure1.2 software was applied to calculate the sizes of CMC/Eu (Ⅲ) nanocomposites. Fourier transform infrared spectra (FTIR) were recorded on a TENSOR27 infrared spectrometer (Bruker company, Germany). Fluorescence spectra were recorded on a FluoroMax-4 fluorescence spectroscopy (HORIBA Scientific, Japan). The excitation was at 370 nm, and the scan wavelength was in the range of 450–650 nm. UV absorption spectra were recorded on a UV Spectro-photometer (UV-6100A) (Shanghai Yuanye Analytical Instrument). The fluorescence lifetime was measured by FluoroSENS fluorescence spectrometer (GILDEN Photonics, Clydebank, UK).

#### 2.2.2. Preparation of CMC/Eu (Ⅲ) Composite

The EuCl_3_ solution and CMC/Eu (Ⅲ) nanocomposite were prepared according to the literature [24]. Concentrated hydrochloric acid was dropped into Eu_2_O_3_ powder in a beaker very carefully. Then, the beaker was put into a hot water bath to evaporate the unreacted hydrochloric acid order to obtain EuCl_3_ solution with the concentration of 0.0346 mol/L [24]. The EuCl_3_ solution was added into the CMC solution slowly. The reactants in 19:1 of the mass ratio of CMC to Eu^3+^ were reacted at 70 °C and pH 7.0 for 35 min. Then, the suspension was dialyzed in deionized water until no precipitation of AgCl, when AgNO_3_ was added. Finally, the suspension was dried in oven at 70 °C to obtain the CMC/Eu (Ⅲ) nanocomposite [25].

#### 2.2.3. Preparation of Food Simulants Contacting with Different Food-Contacting Paper Materials

According to the Chinese standard GB 31604.18-2016 [20] and GB 31604.1-2015 [26], we prepared 10% ethanol, 4% acetic acid, 20% ethanol, and maize oil as food simulants that imitated waterborne food, acidic food, alcohol beverage, and greasy food (designated as S1–S4), respectively. Then, the paper food bag, paper wrap, paper container, and paper cup were treated with the food simulants according to GB5009.156-2016 [27]. During the process, the paper food bag and paper wrap were cut into rectangular films with an area of 5 cm × 10 cm and then put into the food simulants at 100 °C for 1 h. The paper containers and paper cups were filled with food simulants and then kept at 100 °C for 1 h.

#### 2.2.4. Detection of Acrylamide in Aqueous Solution by CMC/Eu (Ⅲ) Probe

Next, 0.6 mL of acrylamide aqueous solutions were added to 2.4 mL of CMC/Eu (Ⅲ) suspensions (5%), respectively. These mixed solutions were kept for 3 min, and then, the fluorescence intensity (λ_em_ = 615 nm) was detected at room temperature (λ_ex_ = 395 nm). The changes in the fluorescence intensities of the solution in the range of 500 nm–750 nm were recorded.

To detect the selectivity of fluorescent probes, 0.6 mL of 1 mmol/L interference substances (Fe^3+^, Ca^2+^, Ba^2+^, Zn^2+^, Na^+^, K^+^, Cl^−^, Br^−^, HCO_3_^−^, CO_3_^2−^, asparagine, glycine, starch, glucose, citric acid, and acrylamide) were added to 2.4 mL CMC/Eu (Ⅲ) suspension. The mixed solution was kept for 3 min, and then, a 395 nm excitation wavelength was used to detect the change of their fluorescence intensity in the range of 500–750 nm.

#### 2.2.5. Determination of Standard Curves in Different Mediums

Acrylamide solutions with concentrations of 0.50 mM, 0.10 mM, 0.08 mM, 0.06 mM, 0.04 mM, 0.02 mM, 0.01 mM, 1.00 µM, and 0.10 µM were prepared in the deionized water, 10% alcohol aqueous solution, 4% acidic food, 20% alcohol aqueous solution, and maize oil, respectively. The fluorescence intensities were detected in the same way as Section 2.2.4. Then, the relationships between fluorescence intensities and acrylamide concentrations were obtained, respectively.

#### 2.2.6. Detection of Migrating Acrylamide in the Food Simulants

The standard addition method was used in detecting migrating acrylamide in all food simulants in contact with different food-contacting paper materials [28]. First, 10 μM, 50 μM, and 100 μM acrylamide were spiked into 0.6 mL of the simulants mentioned in Section 2.2.3, respectively. Then, 2.4 mL CMC/Eu (Ⅲ) fluorescent probe were added into the simulants at room temperature. After 3 min, the fluorescent detection was carried out at 395 nm excitation wavelength. All samples were measured for five times, and the averages were reported.

## 3. Results and Discussion

### 3.1. Detection of Acrylamide by CMC/Eu (Ⅲ) Fluorescent Probe

#### 3.1.1. The Stability of CMC/Eu (Ⅲ) Suspension

**Figure 1:**
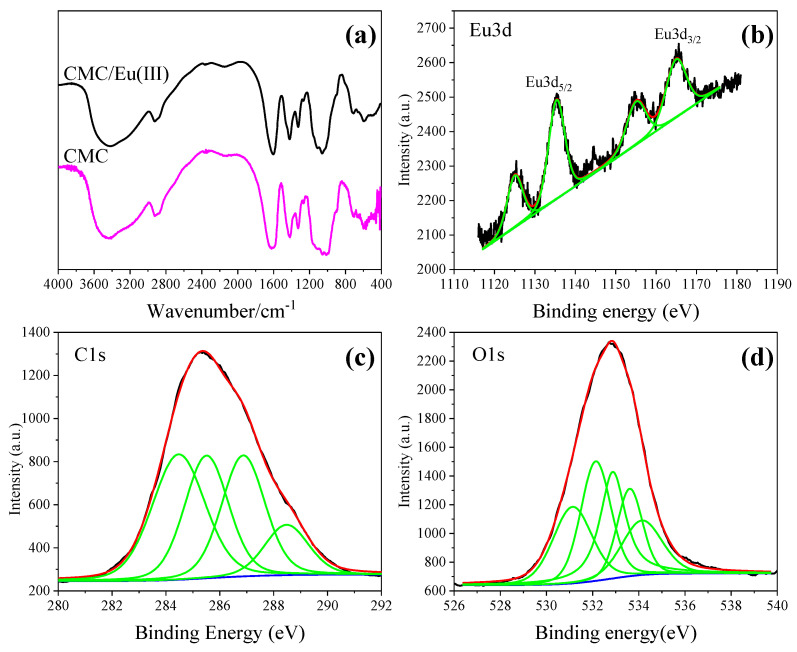
FTIR and XPS spectra of CMC/Eu (Ⅲ) composite. (**a**) FTIR spectra; (**b**–**d**) XPS spectra.

**Figure 2:**
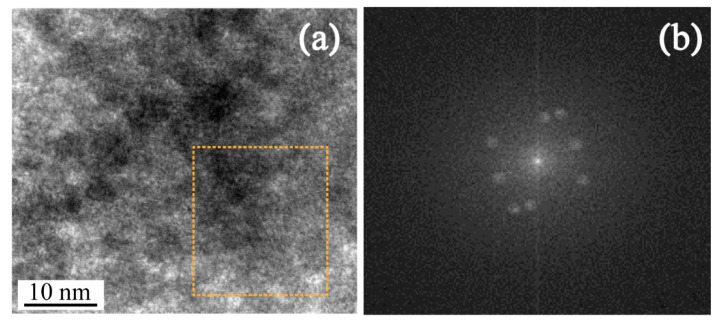
TEM and diffraction patterns of CMC/Eu nanoparticles. (**a**) TEM micrograph; (**b**) the selected area electron diffraction (SAED) pattern.

The CMC/Eu (Ⅲ) composite was synthesized successfully according to the FT-IR (Figure 1a) and XPS (Figure 1b–d) characterization by comparing with those in previous reports [24,29]. The TEM image (Figure 2a) demonstrated the average size of the CMC/Eu (Ⅲ) composites was smaller than 10 nm, and these nanocomposite particles were uniformly distributed. SAED (Figure 2b) showed the diffraction pattern of a single-crystal structure of CMC/Eu (Ⅲ) nanocomposites [24]. These results suggested that CMC/Eu (Ⅲ) nanocomposites were successfully synthesized.

**Figure 3:**
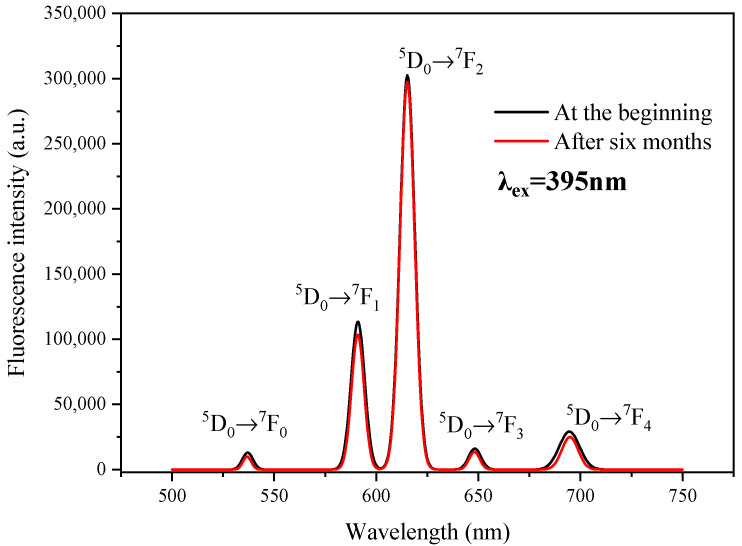
Fluorescence emission spectra of CMC/Eu (Ⅲ) in suspension.

The fluorescence emission spectrum of 5.0% CMC/Eu (Ⅲ) suspension under 395 nm excitation is shown in Figure 3. The spectrum displayed five emission peaks at 537 nm, 590 nm, 615 nm, 648 nm, and 696 nm, respectively, which belonged to ^5^D_0_–^7^F_J_ (J = 0,1,2,3,4) electronic transition of activated Eu^3+^ ion [30]. This indicates that the CMC/Eu (Ⅲ) suspension showed the characteristic emission spectrum of Eu^3+^ ion.

The 5.0% CMC/Eu (Ⅲ) suspension was able to keep hypodispersion, and no precipitation or other changes were observed after six months. Furthermore, the emission intensity of the fluorescence spectrum of the CMC/Eu (Ⅲ) suspension did not decay, indicating that the CMC/Eu (Ⅲ) suspension was highly stable, which benefited the wide application of this fluorescence probe [25]. The great stability and dispersion of CMC/Eu (Ⅲ) suspension were mainly because of the formation of nanoparticles [28].

#### 3.1.2. The Response Time of CMC/Eu (Ⅲ) Fluorescent Probes to Acrylamide

**Figure 4:**
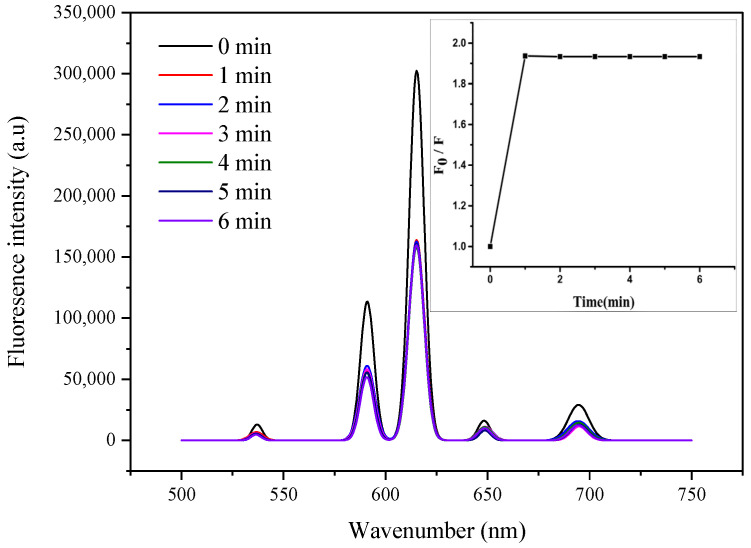
Effect of reaction time on the fluorescence intensity of CMC/Eu (Ⅲ) probe at 544 nm (F_0_, pure CMC/Eu (Ⅲ) fluorescent probe; F, CMC/Eu (Ⅲ) fluorescent probe within 0.06 mmol/L acrylamide).

Figure 4 shows the response time of CMC/Eu (Ⅲ) fluorescent probe for the acrylamide detection. After adding 0.06 mmol/L of acrylamide to the CMC/Eu (Ⅲ) fluorescent probe, the fluorescent intensity significantly decreased in 1 min, reaching a minimum value, and kept unchanged for the remaining 5 min.

The result of Figure 4 demonstrates that the CMC/Eu (Ⅲ) fluorescent probe can significantly and rapidly respond to acrylamide. In our later study, we used 3 min as the detection time to ensure complete reaction and stable peak intensity value.

#### 3.1.3. Selectivity of CMC/Eu (Ⅲ) Fluorescent Probe for Detection of Acrylamide

**Figure 5:**
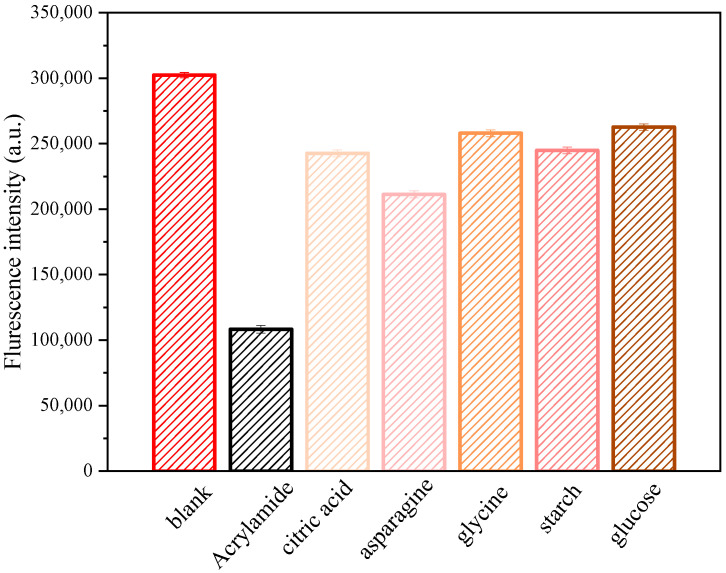
Effect of different substances on the fluorescence intensity of CMC/Eu (Ⅲ) fluorescent probe at 615 nm.

To investigate the selectivity of CMC/Eu (Ⅲ) fluorescent probes to acrylamide, some other substances and ions involved in food and food processing were used to evaluate the interference during the acrylamide detection. The concentration of all these interferents was set at 0.1 mmol/L. Starch and glucose are abundant ingredients in many foods, while citric acid is a common additive in beverage and flavor cakes. Glycine is widely used in foods as a flavoring agent because of its mild sweetness. Asparagine could react by reducing sugars, or other sources of carbonyl produces acrylamide in food when heated to sufficient temperature. Therefore, the substances including asparagine, glycine, glucose, citric acid, and starch were used as interferents. The effects of these substances on the fluorescence intensity of the CMC/Eu (Ⅲ) fluorescent probe are shown in Figure 5. Compared with other substances, only acrylamide was able to significantly reduce the fluorescence intensity of the fluorescent probe.

**Figure 6:**
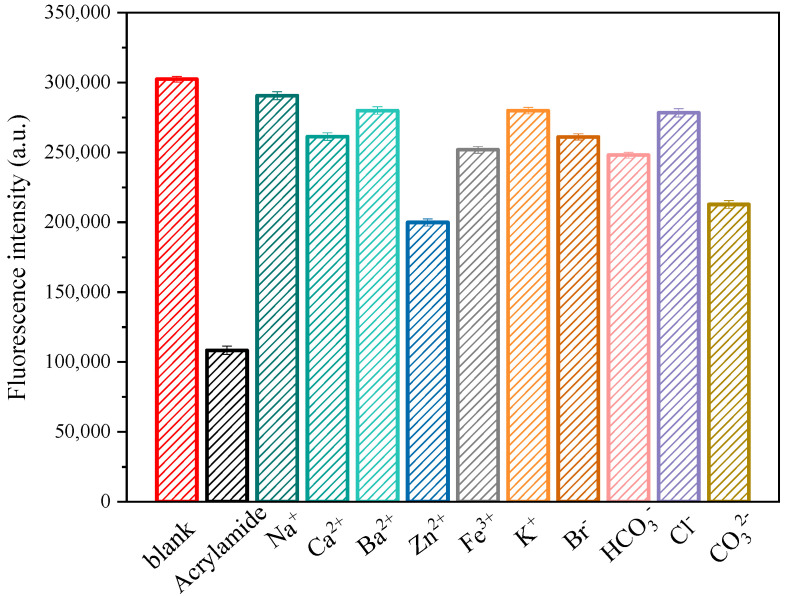
Effect of different ions on the fluorescence intensity of CMC/Eu (Ⅲ) fluorescent probe at 615 nm.

The common ions in food, such as Fe^3+^, Ca^2+^, Ba^2+^, Zn^2+^, Na^+^, K^+^, Cl^−^, Br^−^, HCO_3_^−^, and CO_3_^2^^−^, were also investigated to evaluate their interference on fluorescent probe. As shown in Figure 6, compared to the ionic interfering substances, acrylamide significantly reduced 64.2% of the fluorescence intensity of the CMC/Eu (Ⅲ) fluorescent probe at 615 nm, while other ions showed a limited effect on the fluorescence intensity a reduction of 3.9–33.9%. For example, Zn^2+^ ion reduced the fluorescence intensity of the probe by 33.9%, but addition concentration of Zn^2+^ ions was 13.08 mg/L here, which was much higher than the legal addition concentration of it according to the GB 2760-2014 rules, i.e., 2.4 mg/L, for the use of food additives [31]. These experiments suggested that the CMC/Eu (Ⅲ) fluorescent probe has high selectivity to acrylamide detection.

#### 3.1.4. Effect of Acrylamide Concentrations on the Fluorescence Emission of CMC/Eu (Ⅲ) Fluorescent Probe

**Figure 7:**
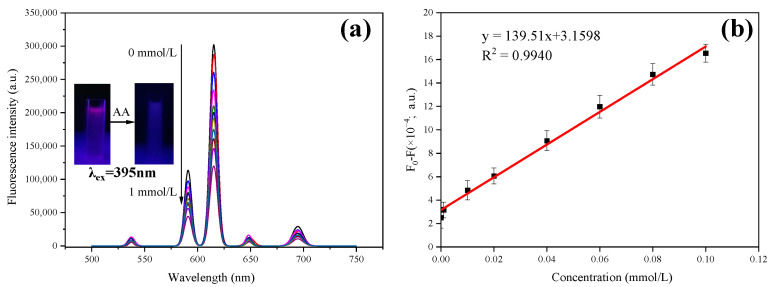
(**a**) Fluorescence emission spectra of CMC/Eu (Ⅲ) in the presence of acrylamide with different concentrations in aqueous solution (0 mM–1 mM). Inset left: photos under UV light at 254 nm. (**b**) The linear relationship between the fluorescence intensity of CMC/Eu (Ⅲ) at 615 nm and acrylamide concentration (0.1 μM–100 μm).

Figure 7a showed the fluorescence spectra of CMC/Eu (Ⅲ) fluorescent probe in different concentrations of acrylamide solution. After adding acrylamide, the CMC/Eu (Ⅲ) fluorescent probe still exhibited the characteristic emission spectrum of Eu^3+^, but the peak intensity decreased gradually with the increase of the acrylamide concentration. When the concentration of acrylamide increased to 1 mmol/L, the fluorescence intensity at 615 nm was down to 19.10% of the initial intensity, which showed that the fluorescence of the probe was gradually darkened from the initial red color under the ultraviolet lamp (Figure 7).

In addition, as shown in Figure 7b, there was a good linear relationship between the fluorescence intensity of CMC/Eu (Ⅲ) fluorescent probe at 615 nm and the concentration of acrylamide when the concentration was in the range of 0.1–100 μmol/L (y = 139.51x + 3.1598; R = 0.9940). The emission spectrum of the fluorescent probe without adding acrylamide was carried out for ten times in deionized water, and the standard deviation of the fluorescent probe was calculated [32]. Then, the LOD of the fluorescent detection for acrylamide was 0.085 μg/L (signal-to-noise ratio 3), according to IUPAC [33].

This LOD was lower than the guideline value of acrylamide in drinking water set by WHO [10]. Compared with other complex, expensive, and technically demanding methods (listed in Table 1 with the LOD) [23,34,35,36,37], the CMC/Eu (Ⅲ) nanocomposite-based fluorescent probe does not require complex analytical procedures and has high sensitivity and selectivity, providing a new analytical method for the acrylamide detection.

### 3.2. Detection of Acrylamide in Different Food Simulants by CMC/Eu (Ⅲ) Fluorescent Probe

#### 3.2.1. Acrylamide Concentrations on Fluorescence Intensity of CMC/Eu (Ⅲ) Fluorescent Probe in Different Food Simulants

**Figure 8:**
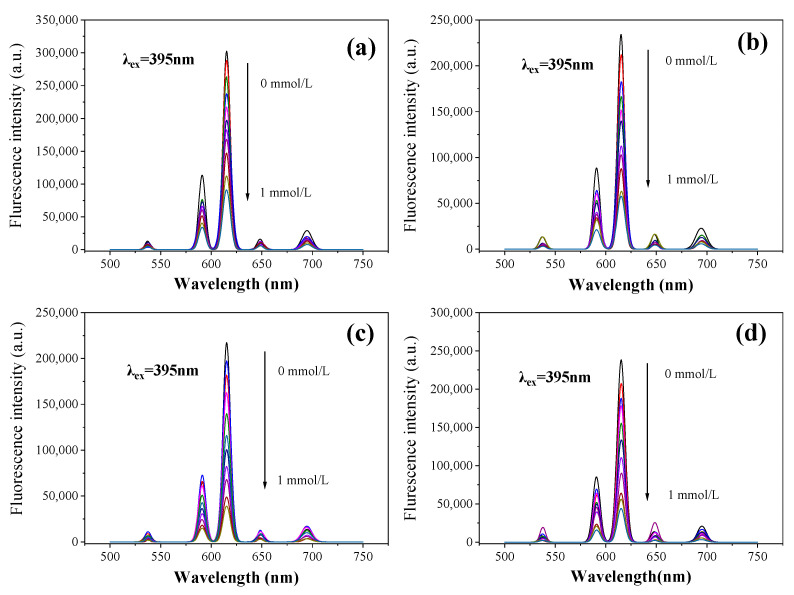
The effect of acrylamide standard solution on fluorescence intensity of CMC/Eu (Ⅲ) fluorescent probe in (**a**) waterborne food simulants, (**b**) acidic food simulants, (**c**) alcoholic beverage simulants, and (**d**) greasy food simulants.

The effects of different concentrations of acrylamide solution on the fluorescence intensity of CMC/Eu (Ⅲ) fluorescent probe in waterborne food, acidic food, alcoholic beverage, and greasy food simulants are shown in Figure 8.

The fluorescence intensity of the fluorescent probe decreased with the increase of the concentration of acrylamide solution in the four kinds of food simulants. When the concentration of acrylamide increased to 1 mmol/L, the fluorescence intensity of the CMC/Eu (Ⅲ) fluorescent probe decreased to the 29.70%, 23.70%, 17.70%, and 17.98% of the initial intensity, respectively.

#### 3.2.2. Linear Relationships and LOD

**Figure 9:**
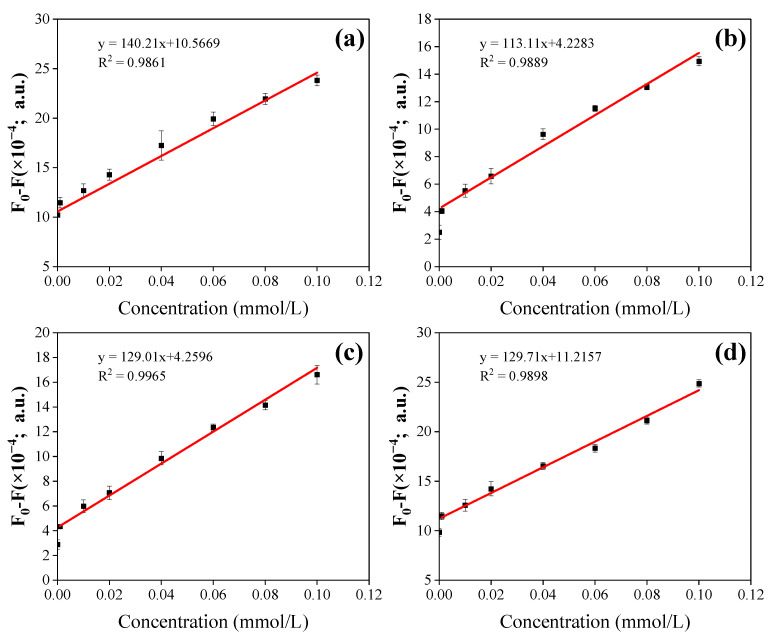
The linear relationship between the fluorescence intensity of CMC/Eu (Ⅲ) at 615 nm and acrylamide concentration in (**a**) waterborne food simulants, (**b**) acidic food simulants, (**c**) alcoholic beverage simulants, and (**d**) greasy food simulants (0.1 μM–100 μM).

Acrylamide standard solution of 0.10 mM, 0.08 mM, 0.06 mM, 0.04 mM, 0.02 mM, 0.01 mM, and 1.00 μM in waterborne food, acidic food, alcoholic beverage, and greasy food simulants, respectively, was prepared. The relative fluorescence intensity of CMC/Eu (Ⅲ) fluorescent probe at 615 nm was measured. The curves of acrylamide concentration versus relative fluorescence intensity were plotted and shown in Figure 9, along with the linear equations, correlation coefficients, and the LOD.

The relative fluorescence intensity of CMC/Eu (Ⅲ) fluorescent probe at 615 nm was well linearly correlated to the acrylamide concentration in the tested samples in the concentration range of 0.1 μm–100 μM. The linear correlation coefficient was from 0.9861 to 0.9965, suggesting a high degree of fitting. The control experiment using CMC/Eu (Ⅲ) fluorescent probe without acrylamide was performed for 10 times, and the standard deviation was calculated. The LOD of the fluorescent detection for acrylamide in different food simulants were 0.22 μg/L, 0.36 μg/L, 0.17 μg/L, and 0.22 μg/L (signal-to-noise ratio 3), indicating a high sensitivity and high dynamic range [38,39].

#### 3.2.3. Measurement of the Migrating Acrylamide from Food-Contacting Paper Materials into Food Simulants

The migrating acrylamide in four different kinds of food simulants from different food-contacting paper materials was measured. The recoveries and relative standard deviations (RSD) of different acrylamide concentrations in all kinds of food simulants from food-contacting paper materials are shown in Table 2. For all the experiments, the recoveries of acrylamide ranged from 97.30% to 108.30%, and the RSDs were all lower than 4%, indicating the high accuracy of this detection method using CMC/Eu (Ⅲ) fluorescent probe. Additionally, the successful measurement of migrating acrylamide under lab condition suggested the CMC/Eu (Ⅲ) fluorescent probe can be potentially applied for real food samples, such as hot tea, wine, vinegar, and deep-fried dough sticks [40].

### 3.3. Possible Mechanism of CMC/EU(Ⅲ) Fluorescent Probe for Acrylamide Detection

**Scheme 1:**
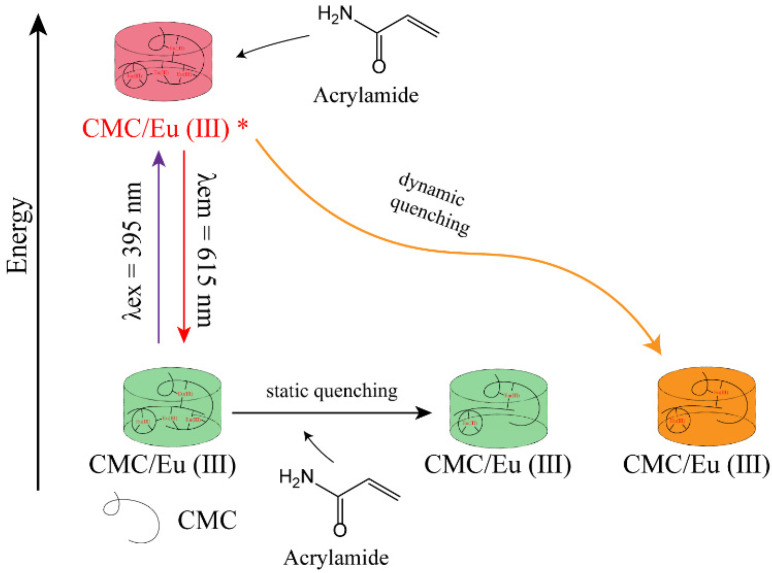
Possible quenching mechanism of CMC/Eu (III) fluorescent probe. CMC/Eu (III) *: the excited state of CMC/Eu (III).

**Figure 10:**
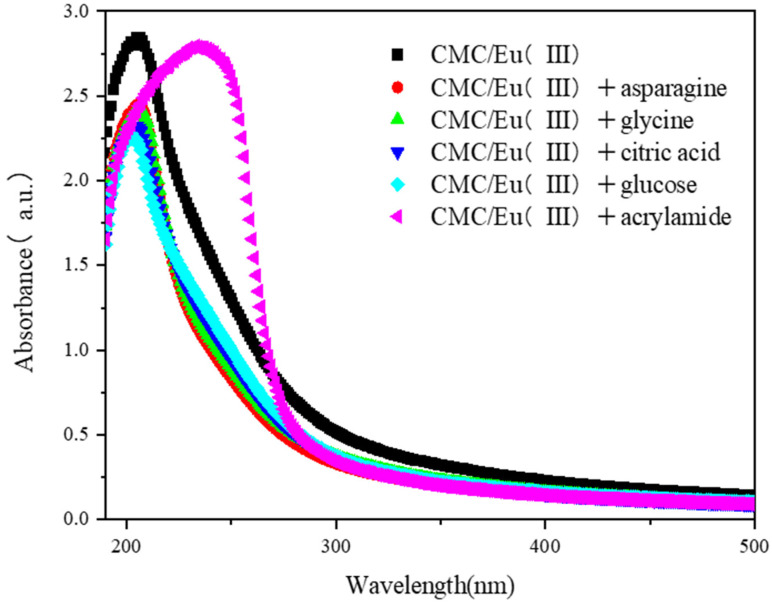
The effect of acrylamide (0.1 mmol/L) on UV–vis spectra of CMC/Eu (Ⅲ) fluorescent probe.

The effect of acrylamide on ultraviolet spectrum of the fluorescent probe is shown in Figure 10. The CMC/Eu (Ⅲ) fluorescent probe has an absorption peak at 205 nm. Adding asparagine, glycine, glucose, and citric acid did not change the absorption spectrum of the fluorescent probe, indicating no interaction between these substances and CMC/Eu (Ⅲ). However, when acrylamide was added to the fluorescent probe, a broader absorption peak shifted significantly from the 205 nm to 234 nm, indicating that acrylamide and CMC/Eu (Ⅲ) formed a new non-fluorescent substance in the static state. Such non-fluorescent substance attributed to the static fluorescence quenching [41]. The presence of a C=O dipole, to a lesser extent than N-C dipole, allows acrylamide to act as H-bond acceptors. The presence of N-H dipoles allows acrylamide to function as H-bond donors as well. Consequently, the O atom in acrylamide could accept H-bonds from water and CMC chains, and the H atoms in N-H could donate H-bonds. Moreover, the O, C, and N atoms in acrylamide have molecular orbitals occupied by delocalized electrons, forming a planar conjugated structure, and the negative charge on the oxygen in an alternative structure by a resonance could function by electrostatic interaction with Eu^3+^ to enhance the conjugation system in CMC/Eu (III). As a result of the non-fluorescent substance developing by electrostatic interaction and H-bonds between the probe and acrylamide, the UV absorption of the probe after adding acrylamide shifts to a long wavelength. Meanwhile, asparagine, an amino acid containing an amide group, could also form H-bonds with CMC to reduce the fluorescent intensity of the probe a little (see Figure 5). However, the interactions between asparagine and Eu^3+^ could be largely prevented owing to its pyramidal bigger volume of asparagine, which hardly moves into the curve chains of CMC (see Scheme 1).

**Figure 11:**
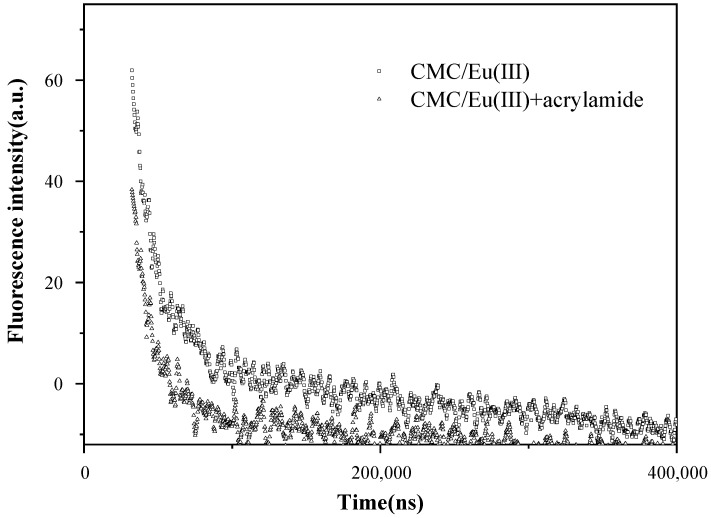
Emission lifetime of CMC/Eu (Ⅲ) probe in the absence and presence of acrylamide (0.1 mmol/L).

Generally, the occurrence of fluorescence quenching might also attribute to dynamic quenching by the reaction of quenchers with the excited-state fluorescence [42]. The effect of acrylamide on the fluorescence lifetime of the CMC/Eu (Ⅲ) fluorescent probe is shown in Figure 11. It can be found that the fluorescence lifetime of the CMC/Eu (Ⅲ) fluorescent probe decreased from the initial 0.16 ms to 0.08 ms after adding the acrylamide, indicating that the excited CMC/Eu (Ⅲ) fluorescent probe interacted with acrylamide, and dynamic quenching occurred [42].

According to Stern–Volmer equation, if F_0_/F equals τ_0_/τ, dynamic quenching occurs [43]. However, in this study, the relative fluorescence intensity (F_0_/F, where F_0_ is the fluorescence intensity of the CMC/Eu (Ⅲ); F is the fluorescence intensity of CMC/Eu (Ⅲ) with acrylamide) was 2.26, higher than the corresponding fluorescence lifetime ratio τ_0_/τ (τ_0_/τ = 2.00). Furthermore, it means that the static quenching plays a critical role in the quenching process [44]. Therefore, the quenching of CMC/Eu (Ⅲ) fluorescence by acrylamide attributed to both static quenching and dynamic quenching (Scheme 1).

## 4. Conclusions

In order to detect the acrylamide migration from food-contacting paper materials, a fluorescent probe based on CMC/Eu (Ⅲ) nanocomposites was synthesized via a simple and green method, which had excellent and stable fluorescent emission. The fluorescence intensity of the nanocomposites was reduced with the addition of acrylamide, and the decrease at 615 nm emission was linearly correlated to the acrylamide concentration from 0.1 μM to 100 μM. Moreover, the nanocomposites had high selectivity and sensitivity for the detection of acrylamide. These characters could serve as a fluorescence probe to detect trace acrylamide. The LOD of the probe in deionized water was 0.085 µg/L, which was able to meet the requirement of the US EPA and the WHO guideline value for acrylamide in drinking water. This fluorescent probe was used to detect the migrating acrylamide from four different food-contacting paper materials. The LOD of the fluorescent detection for acrylamide in different food simulants, such as waterborne food simulants, acidic food simulants, alcoholic beverage simulants, and greasy food simulants, were 0.22 μg/L, 0.36 μg/L, 0.17 μg/L, and 0.22 μg/L, respectively. The recovery of acrylamide ranged from 97.30% and 108.70%, and RSD (n = 5) was less than 4.16%, indicating the high-accuracy stable ability of the quantitation of migrating acrylamide. UV–vis and emission lifetime experiments revealed that the quenching florescence of CMC/Eu (Ⅲ) by acrylamide was mainly due to the static quenching.

## Figures and Tables

**Table 1:** The reports on the LOD of acrylamide.

Probe Type	LOD (mM)	Reference
Hemoglobin–gold nanoparticles modified electrode	0.07108	[34]
A fluorescent sensing method based on acrylamide polymerization-induced distance increase between quantum dots	0.03500	[36]
Chemiluminescence ELISA	0.01860	[35]
Functionalized carbon quantum dots (QDs) fluorescent sensing	0.01848	[37]
Fluorescence determination with an iOS gadget–based digital imaging colorimeter	0.53000	[23]

**Table 2:** Recoveries of migrating acrylamide in S1–S4.

	Paper Container				Paper Cup				Paper Food Bag				Paper Wrap			
	Spiked (μM)	Detected (μM)	Recovery (%)	RSD (*n* = 5, %)	Spiked (μM)	Detected (μM)	Recovery (%)	RSD (*n* = 5, %)	Spiked (μM)	Detected (μM)	Recovery (%)	RSD (*n* = 5, %)	Spiked (μM)	Detected (μM)	Recovery (%)	RSD (*n* = 5, %)
S1	10	10.83	108.30	1.16	10	10.34	103.40	2.25	10	10.17	101.70	1.24	10	9.96	99.60	0.87
	50	50.01	100.02	1.43	50	49.81	99.62	1.60	50	49.58	99.16	1.71	50	50.57	105.70	1.22
	100	99.85	99.85	1.95	100	100.19	100.19	1.37	100	100.21	100.21	1.58	100	100.34	100.34	1.31
S2	10	10.15	101.50	1.91	10	9.73	97.30	1.02	10	10.28	102.80	0.94	10	10.05	100.5	1.64
	50	50.26	100.52	1.43	50	50.03	100.06	2.14	50	50.47	100.94	2.61	50	49.77	99.54	3.02
	100	101.31	101.31	0.98	100	100.07	100.07	1.73	100	99.97	99.97	1.59	100	100.68	100.68	1.39
S3	10	10.54	105.40	4.16	10	9.95	99.50	2.45	10	10.07	100.70	1.86	10	9.84	98.40	0.99
	50	49.76	99.52	2.31	50	51.08	102.16	3.40	50	50.14	100.30	1.25	50	50.32	100.64	1.22
	100	97.42	97.42	1.63	100	99.72	99.72	3.15	100	100.13	100.13	0.92	100	98.25	98.25	0.93
S4	10	9.87	98.70	2.12	10	10.26	102.60	1.99	10	10.13	101.30	3.26	10	10.07	100.70	1.41
	50	50.05	100.10	3.59	50	50.11	100.22	1.28	50	49.97	99.94	1.83	50	50.14	100.28	1.42
	100	101.18	101.18	1.80	100	100.25	100.25	1.52	100	99.92	99.92	1.75	100	98.25	98.25	1.15

## Data Availability

Not applicable.
